# Ethical issues in vaccine trial participation by adolescents: qualitative insights on family decision making from a human papillomavirus vaccine trial in Tanzania

**DOI:** 10.1186/s12910-024-01122-z

**Published:** 2024-11-20

**Authors:** Lucy Frost, Ms Tusajigwe Erio, Hilary Whitworth, Ms Graca Marwerwe, Richard Hayes, Kathy Baisley, Silvia de SanJosé, Deborah Watson-Jones, Kirstin Mitchell

**Affiliations:** 1 London School of Hygiene and Tropical Medicine, Keppel Street, London, WC1E 7HT UK; 2 Nuffield Department of Primary Care Health Sciences, University of Oxford, Oxford, UK; 3 Mwanza Intervention Trials Unit, National Institute of Medical Research, Isamilo, Mwanza, Tanzania; 4 International AIDS Vaccine Initiative, New York, USA; 5 ISGlobal, Barcelona, Spain; 6 School of Health and Wellbeing, University of Glasgow, Glasgow, UK

**Keywords:** Consent, Autonomy, Relational ethics, Paediatrics, Adolescent, Trial, Assent, Tanzania, Vaccine, HPV

## Abstract

**Background:**

Research in children is essential for them to benefit from the outcomes of research but involvement must be weighed against potential harms. In many countries and circumstances, medical research legally requires parental consent until the age of 18 years, with poorly defined recommendations for assent prior to this. However, there is little research exploring how these decisions are made by families and the ethical implications of this.

**Aim:**

To explore key ethical debates in decision-making for participation of children and adolescents in a human papillomavirus (HPV) vaccine trial.

**Methods:**

Semi-structured interviews were undertaken with Tanzanian girls (aged 9–16 years) who had participated in an HPV vaccine trial (*n* = 13), their parents or guardians (*n* = 12), and girls together with their parents (in paired parent-child interviews) (*n* = 6). The interviews were analysed using thematic analysis. Interview data came from a qualitative acceptability study undertaken as part of the Dose Reduction Immunobridging and Safety Study of Two Human Papillomavirus (HPV) Vaccines in Tanzanian Girls (DoRIS) trial.

**Results:**

Girls and parents desired collaborative decision-making, with parents ultimately making the decision to consent. However, girls wanted a larger part in decision-making. Decisions to consent involved many people, including extended social networks, the trial team, media outlets and healthcare professionals and this resulted in conflicts to be negotiated. Deciding where to place trust was central in participants and parents considering decisions to consent and overcoming rumours about trial involvement.

**Conclusions:**

Existing models of decision-making help to understand dynamics between parents, adolescents and researchers but neglect the important wider social impacts and the fundamental nature of trust. Children’s roles in discussions can be evaluated using the principles of consent: autonomy, freedom and information. Concepts such as relational autonomy help to explain mechanisms families use to negotiate complex consent decisions. Whilst interviewees supported the maintenance of legal parental consent, researchers must design consent processes centring the child to ensure that whole family decision-making processes are supported.

**Supplementary Information:**

The online version contains supplementary material available at 10.1186/s12910-024-01122-z.

## Background

**Fig. 1:**
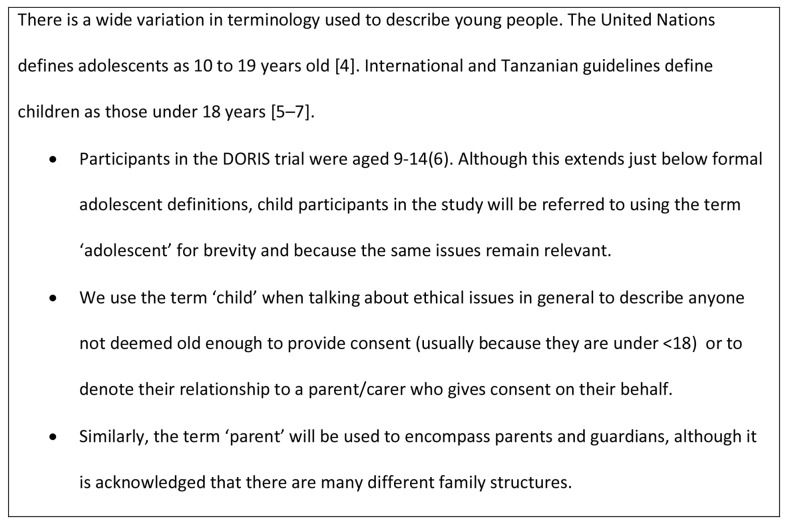
A note on terminology

Balancing the need to protect the safety of children with respect for their autonomy is a widely debated issue in healthcare [1, 2]. Research contexts – including vaccine trials - amplify this complexity because there are uncertainties regarding benefits and risks. In many countries and circumstances, medical research legally requires parental consent until the age of 18 years, with poorly defined recommendations for assent prior to this age [3]. The uncertain status of child and adolescent assent makes it important to understand the processes by which families decide to participate in medical research, including the extent to which the child is involved in decision making, and their opinion given weight. This study draws on interview data from a human papillomavirus (HPV) vaccine trial in Tanzania to explore family decision-making processes regarding participation of adolescent girls in the trial. We first review ethical principles relevant to adolescent vaccine trials and introduce the key issues of decision-making processes and assent. See Fig. 1 for a note on terminology.

### Key ethical concepts relevant to adolescent participation in vaccine trials

Understanding of ethical issues relevant to child participation in vaccine trials relies on several foundational concepts – autonomy, assent and capacity - which are briefly introduced in turn.

Autonomy is a core medical ethics principle [8]. It describes the right of individuals to make informed choices independently [1, 8]. It is often highly prioritised in Western medical ethics due to the individualistic nature of society [9], but has been criticised for not capturing the social influences on decision-making [10].

The legal enactment of autonomy is through consent [11, 12], where a competent individual gives informed permission to pursue a course of action [13]. For children, consent is predominantly legally given by parents as surrogate consent or parental permission [14] although many countries have caveats allowing adolescents to consent in certain circumstances, such as with demonstrated competence [1]. Consent requires individuals to be free to choose, informed about options and to possess capacity for autonomous choice [15, 16]. Information provision should be age and context-appropriate [1, 17]. In paediatric medicine and research, both parents and medical professionals have fiduciary responsibilities to protect the child’s best interests. This can be in opposition to the child’s autonomy, where their wishes are not felt to align with what is best for their health [12]. It is generally accepted that parents will consider the best interests of the family, as well as the child [1].

Assent refers to the process of enabling the child to express their informed opinion on a decision [1], serving both moral and practical purposes [18]. The requirements are less stringent and give space for age-appropriate expression. For example, the absence of strong disagreement may be taken as assent in some contexts [14]. The benefits of this are that it enables the child to be increasingly involved in decision-making, whilst acknowledging the greater vulnerability associated with their stage of cognitive and emotional development. However, it also removes the obligation for assent to be done “well”, and the voice of the young person may remain unheard.

Finally, capacity describes whether an individual has the ability to make a specific decision, and is a widely used construct in medical care [19]. It can be considered a “gatekeeper” to autonomy and consent, as it is required for autonomous choice to be manifested [20]. It is sometimes used interchangeably with “competence”, and sometimes to distinguish between clinical and legal assessments [19]. Capacity is considered on a decision-by-decision basis and it is therefore essential that an assessment is made prior to seeking consent.

Concepts such as “Gillick competence” in the United Kingdom and “the mature minor” in the United States of America (USA) and Canada have developed in recognition that children may be able to make rational decisions about their own health and medical treatment [11, 21].

The concept of relational capacity seeks to overcome the hyper-individualistic conceptualisations that exist surrounding autonomy by situating capacity in its social environment, and promotes dialogue and informational exchange in the face of conflicting views in contrast to “overruling” the child’s perspective [20].

### Application of ethical principles to the trial context

The Nuremberg Code of Ethics sets out the core principle that “the voluntary consent of the human subject is absolutely essential” for medical research [16]. Additionally, the Helsinki Declaration and World Health Organization (WHO) guidelines state that assent should be sought from children where possible, in addition to the legally required parental consent [22, 23]. Notably, WHO guidelines are qualified by saying, whilst legally still assent, there is an ethical argument to consider adolescents’ assent to be “co-consent” alongside parents [23]. Unlike in clinical practice, assent is required in research [1].

Whilst there is a general trajectory of increased competence as children age, this process is not linear, or consistent across individuals or cognitive domains [1, 24, 25]. An important study in the USA considering the role of children in consent processes found that from 9 years old, children could participate in consent processes, and that from age 14 years old competence was equivalent to adults [26]. This is of direct relevance to HPV vaccine trials, which may include children of age 9 years and older because girls aged 9–14 years are the primary target group for HPV vaccination [7]. Contrastingly, other studies have found that even older adolescents struggle to understand assent [27], research purpose [12] and research procedures [28] sufficiently for decision-making. Guidelines must balance respect for emerging autonomy and protection in vulnerability, particularly in the context of adolescence being a time of potentially higher and more inconsistent risk taking [1]. More caution is needed in research than clinical decision-making, due to greater uncertainty regarding risks and benefits and greater acceptability of not participating [29], and because some research concepts may be more difficult to grasp [12].

Self-consent of adolescents for vaccination has been widely explored [30, 31] and exists for some established vaccine programmes worldwide, including for HPV [32, 33]. This exploration has been extended to a range of research contexts, with mixed conclusions regarding appropriateness [24, 34].

Dual consent models in research have been proposed, where consent is sought from both child and parent [35]. Alternatively, some have advocated for models in research where children are assessed for capacity for each decision, and parents are only involved where the child is considered to lack capacity [18, 36]. This perspective dismisses the role of assent which is viewed as poorly constructed and implemented, with no clear accountability.

### Decision-making processes in adolescent trial involvement

WHO research guidelines differentiate between the legal act of consent and the process of decision-making itself, specifying that it is essential that children are involved in decision-making about participation in a study, taking into account their age, prior experiences, maturity and intellect, and their individual and family circumstances [23].

Snethen et al. (2006) conceptualised four levels of involving children in decisions about study consent [37]: Exclusionary - the child is not involved; Informative - the child is informed but cannot affect the decision; Collaborative - the child is actively involved in decision-making, with parents making the final decision; Delegated - the child makes the decision.

They recognised that the parents’ goals and perception of their roles and the child’s involvement were all important in determining balances of involvement and power. It was also recognised that decision-making often involves a wider spectrum of individuals, such as extended family; this is under-recognised in research [37]. Others have emphasised that family type and culture profoundly influence decision-making strategies [38].

It is also important to consider what people want from decision-making and consent. One study looking at a HIV vaccination trial in South Africa found that, whilst some felt that adolescents should be able to consent independently, many adolescents and parents preferred parents to make the official decision regarding consent. For the adolescents, this related to wanting the reassurance of parental support for ongoing study engagement, such as transportation, as much as decisional support [39]. Other studies have also found that many adolescents and parents were happy to retain parental consent models [40].

It has been argued that many conceptualisations of children’s decision-making are viewed through a narrow, cognitive-framework, that concludes that they are cognitively lesser than adults [19]. They argue that wider conceptualisations that included beliefs, values and a recognition of the impact of adults on children’s ability to make decisions would enable children to be valued more highly for what they bring to decision-making processes. Practically, these narrow conceptualisations mean that adults are assumed to have capacity and therefore be able to make decisions, whereas children must work harder to achieve decision-making status [20]. There is a need for decision-making models that explore emotional and values-based factors and situate decision-making capacity in its social environment [41].

### Negotiating consent in a socio-cultural environment

Decisions around adolescent trial involvement require negotiation between multiple actors, each considering an array of factors and interests [42]. Geissler et al. discussed the relational nature of ethics in a vaccine trial in The Gambia [43]. They describe a blurring of the discrete lines advocated for in international ethical codes, and instead described ethical practices that relied on familiarity, ambiguity, and engagement to navigate dynamics of power and transactionality.

A vaccine trial study in Kenya emphasised that community-level involvement was essential for successful research engagement and should be established before approaching individuals [44]. Other people’s concerns appeared to influence likelihood of consent and trial completion, and seemed to be linked to conflicts within the community as well as specific worries about enrolment. These were often manifested in the form of rumours about vaccination [44].

The Tanzanian Guidelines of Ethics for Health Research remark that elders, community leaders and husbands may have perceived authority in decision-making at a family or community level. It recommends that researchers should, in these cases, emphasise best interests decision-making over autonomy [45].

The appropriateness of a single international concept of consent has been contested, with some arguing the need for localised, socially and culturally informed definitions [46]. For example, there is often a prioritisation of individual autonomy in Western cultures, that may not translate as well to all regions and may impact the interpretation of ethical principles [47]. Issues of assent and consent have been more extensively explored in high-income countries, potentially omitting important considerations for low-income countries. For example, Cheah and Parker reflected on the difficulties obtaining assent in the context of pre-existing parental consent in a study in Bangladesh due it being less acceptable to disagree with ones’ parents [18]. They also raised potential issues of illiteracy, earlier maturity, differential education across generations, less familiarity with medical research, alternate family structures and regulatory challenges. Clearly, all countries are heterogeneous and the precise opportunities and challenges need to be considered within local contexts. However, an evaluation of consent for a vaccine trial in The Gambia found that, when sensitively implemented with respect to cultural context, an international definition of free, autonomous and informed consent can be successful [15].

### How this research will add to what is known

Our understanding of the complex processes of family decision-making is limited, particularly in the context of decisions regarding adolescent trial involvement and within different social environments. Conceptual models that apply an ethical and social perspective have value in increasing our understanding of how and why families make decisions to be involved in adolescent trials. This can inform and deepen our understanding of key ethical debates in adolescent trial involvement, which can be applied to improving public health research and practice.

The data presented here were made available from the Dose Reduction Immunobridging and Safety Study of Two Human Papillomavirus (HPV) Vaccines in Tanzanian Girls (DoRIS) trial. The DoRIS trial was an unblinded randomised clinical trial comparing the immunogenicity and safety of one versus two or three doses of two different HPV vaccines in healthy Tanzanian schoolgirls aged 9–14 years. The DoRIS trial took place in the Mwanza region of Tanzania. Mwanza is a city located on the shore of Lake Victoria and surrounded by rural areas. Methods and main results have been described previously [7, 48] In brief, 930 girls were randomised to receive one, two or three doses of Cervarix^®^ or Gardasil^®^. All participants were followed up to 36 months post-vaccination, with blood collected pre-vaccination and at months 1, 7, 12, 24 and 36 for measurement of immune responses to the vaccines. Girls in the two and three dose arms are continuing in follow-up to 9 years post-vaccination.

A qualitative ancillary study explored (a) the acceptability of the HPV vaccine and dose (published separately [49]); and (b) how families made decisions around consent/assent. The latter study aim is the basis of this paper.

Specifically, the aim of this study was to explore key ethical debates involved in decision making for adolescent participation in an HPV vaccine trial through analysis of interview data.

## Methods

The study comprised individual semi-structured interviews with girls and (different) parents; and paired semi-structured with girls and their parents. In total 31 semi-structured interviews were conducted and examined using thematic analysis. A priori theory was deliberately avoided to remain close to the data, with theory generation occurring throughout the process, drawing deeply on interview data and a conceptual model that was iteratively developed.

### Participant selection and data collection

Data were collected as part of a qualitative study nested within the DoRIS trial between November 2017 and December 2018 [7], that explored the acceptability of different HPV vaccine dose regimens. Methods for the sub-study have been described in detail previously [49]. In brief, eligibility was based on completing the allocated vaccine course and attending a 6-month follow up visit. Random sampling was undertaken within each of two age groups (9–11 and 12–14 years old at the time of vaccination) and each trial arm (1, 2 or 3 vaccine doses of Cervarix or Gardasil-9), and then reviewed to ensure adequate variation across other variables such as religion, tribe and location. Contact with potential participants for the qualitative study was made by the DoRIS team and subsequently by a qualitative researcher, who arranged a meeting to discuss the qualitative sub-study. If the parents and girl indicated that they were happy to be involved, a further date was arranged for the qualitative study interview. Written parental consent and participant assent were undertaken prior to commencing the interview [48].

The interview guide was developed by the Acceptability research team, initially in English, then translated and back translated into Swahili by a Tanzanian member of the DORIS trial. The questions were designed to address questions on acceptability as set by the larger vaccine trial.

### Data analysis

Data from anonymised transcripts were coded using NVivo. An inductive reflexive approach to thematic analysis was used as described by Braun and Clarke [50], comprising the following phases:

1. Data familiarisation - transcripts were read to understand content and to consider emerging connections and concepts;
2. Code generation - application of data-generated codes;
3. Theme construction - development of provisional themes from codes through recognition of common or important ideas;
4. Revising themes - further review of provisional themes to ensure depth and reduce overlap. This included exploring deviant cases to try to better understand why these did not fit provisional themes. These were reported or used to amend provisional themes;
5. Defining themes - definition of scope and content;
6. Producing the report - final stage of analysis where themes were checked through writing up and comparison with other literature.

Thirty-one interviews are a substantial number in qualitative research, and the main themes were sufficiently aligned to suggest proximity to data saturation (the point at which no additional insights are generated in further interviews); this helps improve the validity of the themes generated [51]. Anonymised quotations from the interviews were used to illustrate findings and improve reliability. This also provided transparency into how themes were arrived at.

### Ethical considerations

Ethical approval for the DoRIS qualitative study was granted from the London School of Hygiene and Tropical Medicine Ethics Committee (ref: 11972) and the National Health Research Ethics Committee in Tanzania (ref: NIMR/HQ/R.8a/Vol.IX/2682).

### Informed consent

Consent for participation in the acceptability study was obtained by a qualitative researcher explaining the study, providing written information, and answering any questions. Written consent was obtained from the parents, and written assent was obtained from the girls. Witnessed consent and signing with a thumbprint was obtained in the case of parent/guardian illiteracy. Researchers were additionally trained to be sensitive to signs of reluctance at recruitment or during the interview. No financial incentive was provided. Parents were offered compensation for travel costs, and girls were provided with transport to the study clinic if needed.

### Confidentiality

Data were analysed and stored in the form of anonymised interview transcripts that did not include any participant identifiable data. Risk of deductive disclosure was low, given the nature of the data, but care was taken to ensure that illustrative quotes were free from this risk.

Data were stored on a secure network drive in line with data storage requirements of the University of Glasgow (lead institution for the qualitative acceptability study) and LSHTM.

## Results

Individual interviews were conducted with 13 girls who had participated in the DoRIS trial and with 12 parents of participants. Interviews were also conducted with 6 pairs of participants and their parents, who were different participants from those in the individual interviews. Participant characteristics have been published previously and are presented in Table 1 [49]. Interviews were undertaken at participants’ homes or at a study clinic, according to the participant’s preference. An interview guide was developed as a basis for this (see Supplementary Files 1, 2 and 3). They were audio-recorded with consent, transcribed and translated from Swahili to English.

**Table 1:** Participant characteristics

	GIRLS (*n* = 19)	PARENTS (*n* = 18)
**Type of interview**		
**Individual**	13	12
**Paired**	6	6
**Age in years**,** Median (range)**	12 (9–16)	44 (28–72)
**Gender**		
**Male**	0	4
**Female**	19	14
**Residential setting**		
**Urban**	15	17
**Peri-urban**	4	1
**Religion**		
**Christianity**	16	14
**Islam**	3	4
**Current school level**		
**Primary school**	12	–
**Secondary school**	7	–
**Education Level of parent**		
**Primary School**	–	14
**Secondary school**	–	2
**Vocational training**	–	1
**University**	–	1
**Occupation of parent** ^**a**^		
**Vendor**,** salesman/woman**	–	6
**Farming**,** agricultural work**	–	4
**Housewife**	–	3
**Business man/woman**	–	3
**Unemployed**	–	1
**Other**	–	5
**Number of HPV vaccine doses received**	Girls	Daughters
Empty Cell		
**3 doses**	5	7
**2 doses**	7	5
**1 dose**	7	6

a Some parents gave more than one occupation

In the parental interviews, 7 mothers, 1 father, 1 stepfather, 1 aunt, 1 cousin and one guardian were interviewed. In the paired interviews, 3 mothers and 3 fathers were interviewed. In one of these the father joined part way through the interview also.

Parents and girls’ experiences of the decision-making processes involved in DoRIS trial participation included perceptions of ultimate parental choice and aspirations for consensus-seeking and bilateral involvement. Parents enjoyed the centrality of their role, whereas girls wished for deeper involvement and tried to enact this in a variety of ways. Strategies were employed to balance information from a wide range of sources and try to engage in meaningful decision-making. Trust and distrust were central in how participants and families placed weighting on information. These processes were situated in relationships and the social environment.

### Contested and uncontested autonomy

#### Primary decision-making practices

With the exception of two respondents (both girls) all respondents felt that the final decision to consent rested with parents. The two girls felt that, in the trial consent process, they were the main decision-makers but acknowledged that was alongside their fathers’ decision. Often, it was clear that the girl’s opinion directly informed the “official” parental choice:

Paired 4 (father): Before participating I came with the girl here at Medical Research; she was asked questions by the doctors, and they asked me too. So we agreed. The girl was asked and she agreed; if she had refused, she wouldn’t have participated.

However, the understanding of the final decision resting with the parents did alter dynamics: involvement of girls became optional, and parents’ ultimate decisional authority was assumed.

Girls justified why it was “right” to listen to parents, including that parents were elders, their mothers gave birth to them, they lived at home, they might need parental help and that parents had greater life experience, as well as recognition that parental consent was legally necessary.

Girl 7: It is my duty to listen to my parents and if I go against their wishes they would curse me, and the curse of a parent is strong.

There were examples of parents’ roles being questioned, but these were few. One girl suggested she should be allowed to consent herself because you are “creating yourself in deciding if whether you want it or you do not want it” (girl 2). Another parent felt that they should only advise and let their daughter make the decision:

Paired 3 (father): The best way is [not for] the parents to decide but to discuss and involve the [girl] because the parent’s task is only to advise.

Most girls did not want to consent without their parents being involved. This appeared to be a combination of reassurance in the current consent processes and a feeling that parents should have the right to make these decisions.

One parent strongly felt that to allow girls to consent themselves would make the trial unethical: “Why vaccinate the child without [parent’s consent]? Ee, if it were so, we would conclude that this study is a fraud and unethical.” (paired 1, father).

Parents thought their role was largely to coordinate the process and to make the final decision. This included choosing who was involved and in what capacity, and then weighing up the information.

Girls’ roles were more contested than parents’. All girls except one felt they should be involved in decisions regarding them. Experiences varied: many girls reported never being asked about decisions regarding themselves, but most felt they were involved in the trial decision to some degree. Involvement ranged from parents informing girls of pre-made decisions, to consensus-seeking family meetings where girls were encouraged to advocate for their wishes: “He was giving me his views and I was giving him mine until we decided to take part” (paired 3, girl).

Some participants raised how gender influenced decision-making patterns, although mechanisms were not ubiquitous. Some families described dynamics in which the mothers did much of the information collection and synthesis and provided recommendations to the father.

Parent 6 (mother): I was the one who made the decision. After attending the seminar, and had detailed information about the vaccine, I then decided all alone then I came and talked to her dad and he had no problem about that.

This pattern was also seen across other household decisions, such as getting new school uniforms. However, it was not universal; some fathers played active roles in attending seminars, overcoming other family members’ concerns, and directly engaging with the trial team.

There were only two cases where both parents weren’t included in a decision. In one case, the mother, who lived with the child and father, was excluded from the decision making. In the other case, the father, who did not live with the child and mother, was not included in the process. The girls were unsure why the decision to not include a parent in the consent process was made.

Other, mostly female, members of the extended family were often involved in the discussions, particularly if they lived in the same household. These included girls’ grandparents, aunts, siblings and cousins. The roles played by these individuals ranged from surrogate parental figures in the consent process, to being sources of advice.

#### Expressions of autonomy

Girls sought opportunities to influence decisions through reminding parents to attend meetings, strategically discussing issues and involving others. One girl sought her aunt’s support prior to speaking to their father about the trial.

Family structures that didn’t have two biological parents provided girls opportunities for increased agency. Guardians who were not biological parents tended to be more cautious about exerting their own agency, letting girls play larger roles. One guardian seemed to feel particularly responsible for not pressing her own opinion: “I said that because this vaccine is a trial one, what if it affects her and she is not mine…what will I do?” (parent 3, guardian).

Parents also felt age influenced how they weighted girls’ opinions, due to perceptions of cognitive development and decision-making maturity.

Paired 1 (father): It is because she is still in the foolish age. She is not in any position to get to decide until we first get to decide.

Girls recognised that there were limits to their power in these relationships: “you cannot force parents who have refused” (Girl 11), and there were examples of girls choosing, hypothetically, not to pursue their own wishes because “dad said so” (Girl 13).

### Negotiating conflicts and alignment

Largely, trial decisions were made using the template of other decisions, such as schooling. Girls who felt they had been more involved in decisions in the past, also felt that they were involved in decisions around trial involvement. The opposite was also true, with other girls feeling that they hadn’t been involved in either trial or other life decisions.

Disagreements between parents or between parents and children inevitably occurred. Where this happened, it was often approached by trying to convince each other, with a decision made after consensus was reached. Convincing was often done under the framing of “education” or “explaining”.

Parent 12 (stepfather): After I went to school […] I came back home and involved my partner but due to the fear that she had, I had to educate her […] she understood, and she received it well and so I signed the form.

Non-confrontational strategies were preferred, and references to “collisions” (parent 2, father) and “punishment” (paired 1, father) were uncommon. There were hypothetical examples of where limits were set hierarchically, and opinions overruled. When asked what she thought her father would have done in the situation where her mother didn’t want the girl to participate in the trial but her father did, she replied:

Girl 11: On the day that they were coming for me, dad would have told them that her mother has refused and that she should not take part.

However, it was clear that consensus-seeking was almost exclusively seen as the most positive way to make decisions.

Paired 4 (father): Sometimes there can be misunderstandings between the mother and the father. But if you love each other, you sit down together.

### Influence and trust

#### Deciding how to be influenced

Children and their parents strategically engaged with people and resources outside of the core decision-making group. At times it was felt that this information was helpful and was actively sought. Other times it was deliberately avoided, to prevent complicating the decision or to minimise inaccurate information:

Parent 4 (mother): In some families you will get crazy opinions such as “these vaccines are this way or that way, they will do this or that to your daughter” […] Before involving someone, you must first understand them, even if it is your relative.

Information and opinions were received from a wide variety of sources, including the trial team, healthcare professionals, media outlets, community leaders, school lessons, religious leaders and friends, neighbours and family.

Many girls and parents commented that they only felt able to participate in the DoRIS trial because many others were participating: “Another reason is that I wasn’t the only one who went to be vaccinated. We were many of us and all of us saw how important it was to be vaccinated” (Girl 2). The effect of being part of a large group allowed other barriers to be overcome, such as being told it was risky.

Written information distributed by the DoRIS team was mentioned frequently, and seemed to play a unique role in grounding decisions, opening discussions and allowing other opinions to be overruled:

Parent 4 (mother): I asked her whether she would like to go on; and I gave her the pamphlet to read; after reading it I asked her if she still wanted to go on.

Whilst both girls and parents were influenced by a variety of sources, girls tended to get most of their information from their parents and the DoRIS trial, and relied less on other sources, compared to parents who collated information from a wide range of sources. In response to a question about whether one girl had considered anything in particular when contemplating trial involvement she responsed “I was just listening to mum’s opinions” (Girl 3).

Throughout the interviews were examples where uncertainties or inaccuracies in understanding of elements of the trial were apparent. This confusion was most pronounced in the girls but extended across parents as well. This was not necessarily seen as problematic to the participants, who appeared to have their informational needs met. It was unclear whether this related to an initial lack of comprehension, although participants or their parents were required to pass a ‘test of understanding’ prior to participation in the trial [48], or whether it was related to these interviews having occurred months after the consent process.

Importantly, parents did seem to have a good understanding of the consent process, and unanimously seemed to recognise that trial involvement was voluntary: “It wasn’t compulsory. It was an optional thing. It just depends if you like it and no one was forcing you” (parent 9, aunt).

Most respondents were happy with the level of information they had received: “[We had enough information] because they had explained to us and told us that, “if you do not understand, you ask a question”” (Girl 1). Where information was felt to be lacking, various strategies were employed. Many reported that they could return to their information sheets. Several of the fathers reported assertive engagement with the researchers to obtain answers to questions.

Parent 2 (father): We wanted explanation on that, and I actually asked about it twice. Then that guy told me, “You have already asked about that question” but I told him that I have not clearly understood it.

In contrast, many of the girls reported not asking questions they had, and one of the mothers had hoped that peers would ask her question: “I didn’t understand, I wanted to ask and then I said let me wait, maybe there is one of my peers who thinks like me but I didn’t ask.” (parent 9, aunt).

#### Trust and decision-making

Trust played a key role in shaping which external influences became meaningful in family decision-making processes.

Participants used trust as a mechanism to ignore rumours circulating about the trial (including that the vaccine reduced fertility, that it was part of terrorism or Freemasonry, that too much blood would be drawn and that they were planting bacteria into the participant’s body). Deciding to participate required more than just a cognitive balance of factors, but also a trust in people and groups. Certain groups were considered trustworthy, and their opinions were weighted more heavily.

Girls considered their parents amongst the most trustworthy sources, resulting in respect for their opinions even when they didn’t always agree.

Girl 4: [I value Mum’s opinion most] because she was the one that gave birth to me and the one I listen to the most.

Whereas the beliefs of extended family carried weight, the ideas of neighbours, friends and other community members were more complicated. Several people felt it was best to collect as much information as possible, whilst others felt that many people were uninformed, and it was better not to ask them:

Parent 3 (guardian): You know that there are some friends that you can tell them and they end up misleading you. Such a serious matter requires independent thinking.

An exception to this was individuals with lived experience of illness or vaccination. A girl who had received the HPV vaccine and subsequently had a child was given as evidence that fertility rumours were unfounded, and distressing stories of those affected by cervical cancer were used as motivation to trust healthcare and trial institutions:

Parent 7 (mother): There is a girl who has once been vaccinated in this study […] and she have gave birth so after I have met her I then realised that these are only peoples’ words that are […] in the streets.

Healthcare professionals, from within and outside the trial, were considered a trustworthy source of information. One parent had deliberately sought advice from one of her employers who was a doctor, and another had asked a doctor who attended the same church:

Parent 1 (mother): I went to that doctor and asked her […] She told me that thing is fine it has no problem […] when that doctor gave me that certainty, I got peace.

Whilst greatly respected in many ways, religious leaders were not considered to be trustworthy sources about the trial. Many participants felt they might be poorly informed, potentially resulting in inaccurate advice being given:

Parent 3 (guardian): He is not a professional on the side of health, he is a religious leader, and he doesn’t know what’s going on.

Where trusted individuals gave differing opinions, advice against participation appeared to carry more weight.

### A new conceptual model of decision-making

**Fig. 2:**
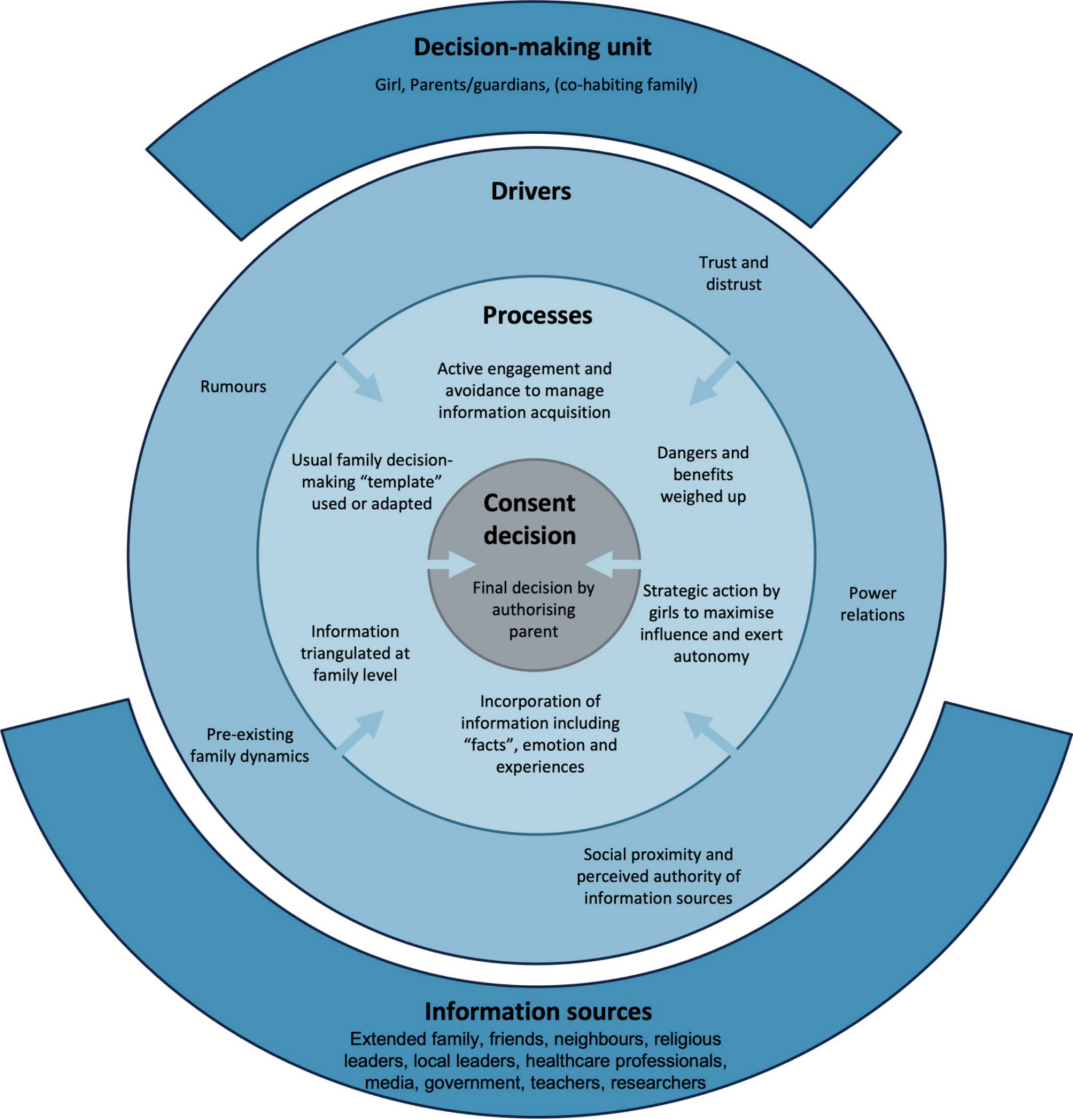
Conceptual model of family decision-making for adolescent trial involvement

The decision-making processes described by the interviewees can be viewed diagrammatically (see Fig. 2). This demonstrates the wide range of factors that influence consent decisions, spanning from governmental trust to small family discussions. Whilst parents, and often one parent, have the authority to make final decisions, the process informing this often involves the child and other parents cooperatively. Complex negotiations of power, social proximity and trust inform consideration of different peoples’ opinions. Parents and adolescents are active agents in this process, asking questions and strategically deciding how much information to seek and where from.

This model builds on previous models by situating decision-making in the wider social context and acknowledging the role that power and trust play in how this information is managed. It recognises that information is not unidirectional but that there are constant negotiations between many actors over time. Finally, it incorporates important aspects of relational autonomy, including the interpersonal nature of decision-making and that decisions are made not solely as logical conclusions after balancing the risks and benefits, but also incorporating emotion, experiences and power dynamics [10, 20, 52]. It is expected that this model would be useful in exploring processes across cultures, rather than exclusively Tanzanian contexts. However, it is likely that the weighting placed on various factors would vary significantly, and that there may be additional factors not identified here.

## Discussion

The DoRIS qualitative interviews demonstrated that both girls and parents recognised the value of each other’s opinions in the decision-making process. For parents, decisional authority was assumed, whereas girls described their role as more contested. Both groups tried to navigate the decision alongside each other, although generally it was agreed and desired that parents made the final decision. These processes were not based purely on objective facts, but were founded on trust, pre-existing family dynamics and a wider social context. This raises important ethical questions about how to view the concept of autonomy for the girls, what constitutes informed consent and how this can best be negotiated in the context of adolescent trials.

### Understanding the findings in the context of existing decision-making models

Many characteristics of decision-making identified are supported by other studies. The idea that parents have ultimate decisional authority is common [15, 53], although not universal [37]. Similarly, age is often felt to be associated with access to and responsibility in decision-making [53, 54] although developmental research suggests that age is only one component of capacity to make decisions and is not always linearly associated [12, 25]. Finally, consensus-seeking with some level of parental and child involvement is a common strategy [55].

Snethen et al. proposed the exclusionary, informative, collaborative and delegated decision-making model [37]. These approaches vary in the parents’ goals from interaction, the type and extent of the child’s involvement, and the parents’ idea of their role. Different families will choose different approaches, based on factors such as pre-existing dynamics and the impact of the decision in question [37, 38]. Applying this framework to the interview data illustrates that informative and collaborative approaches were used primarily, with some infrequent descriptions of exclusionary decision-making. Notably, Snethen’s model suggests parent-led dimensions, but the interviews here suggest that girls have their own ideas of how their involvement should look and take steps to manifest this. This role is not recognised in the model, and there is no exploration of how conflicts in perceptions of role might be managed.

Pre-existing family dynamics were a large contributor to how decisions about trial involvement were made, according to the interviews. Families that described more collaborative decision-making in other life decisions were more likely to describe collaborative decision-making around trial consent, a finding recognised elsewhere [37, 56].

Detailed conclusions about gendered roles were precluded by the available data and could usefully be explored further. Whilst both parents were involved to some degree, mothers seemed to have a more process-orientated involvement, with fathers providing the final authorisation. A study in The Gambia also found that mothers tended to be more involved than fathers in the decision-making, although this did not necessarily mean that they had the ultimate decisional authority [15].

Other studies have found that parents felt that, whilst other people were useful for advice, the main decision lay with them [15]. Whilst this also tended to be the case in our interviews, there were multiple examples of where extended family members living under the same roof were heavily involved in decision-making alongside parents and children. When family members were not living together, opinions were sought in a more advisory capacity. Others have also suggested that the role extended family members play is often overlooked [37].

### Who gives consent?

Adolescent self-consent is in place for vaccine programmes in some areas of the world [31, 57] and there has been exploration of the appropriateness of this for research participation [24, 34]. However, the girls did not appear to want, nor feel comfortable with, legal consent without parents. Combined with some girls being as young as 9 years old, below most estimates of adequate cognitive development for healthcare decisions [26], these interviews did not suggest that self-consent for the vaccine trial was appropriate or desired. However, there was a great desire from both girls and parents to have meaningful conversations about the decision. It is therefore worthwhile exploring the underlying assumptions of informed consent from the perspective of adolescents, as it gives a more nuanced insight into the role that adolescents can and do play in the decision-making process and provides opportunities for future trials to build consent processes that support this.

In common ethical conceptualisations, consent needs to be autonomous, free and informed [16]; these criteria will be examined in turn.

#### Autonomy: individual or relational?

It was clear from the interviews that decision-making was a co-produced process between parents, children and, at times, a more extended network. The most commonly cited model of autonomy described in core ethical texts, the in-control agent model, focuses on the person’s ability to weigh up information to come to an individual, logical conclusion, with the ultimate aim of independence [8, 9]. This model has been criticised for being hyper-individualistic and overly rational at the expense of social-embeddedness, emotion, and embodied experience [10]. The life worlds of adolescents are embedded in their family and social life and the idea of making a completely individual decision may imply an arbitrary and damaging division. This is demonstrated in the interviews, where it is difficult in some ways for the adolescents to draw a line between where their own opinions stop, and their parents’ opinions start, a pattern also seen elsewhere [9]. Additionally, themes of fear and trust that were borne out in the discussions of rumours and trusted authority clearly had heavy influences on how decisions were made, not only through rational decision-making but also through embodied decisions of who was “trustworthy” and through the emotional experience of making decisions in the face of concerning information. A relational model allows us to see adolescents as autonomous in a way that an in-control agent model does not as the criteria it sets are more applicable to adolescents. The benefit of this is that adolescents become legitimate (relationally) autonomous agents, whose opinions are an essential and therefore uncontested component of the decision-making team. However, relational autonomy models recognise that there is the potential for exploitation through oppressive relationships, and this can be detrimental to emerging autonomy [9].

Relational autonomy allows the inclusion of other family members in the conversation. Decision-making in paediatrics can impact the whole family [37] and parents play integral roles in supporting children’s thought processes and competence [58], but are often neglected in in-control agent models of autonomy. The extent to which parents include children in decision-making in general will also influence their ability to make decisions about research [12], consistent with our data demonstrating that general family decision-making was used as a template for consent decisions. This also goes some way to mitigating concerns about potentially higher risk taking that can be seen in adolescence [1].

#### Freedom of choice in adolescent trial consent

The concept of freedom of choice in relation to adolescent involvement in consent is a complex one. Legally, adolescents do not have the freedom to choose as consent lies with parents and this knowledge underpins the discussions around choice in the interviews. Further, there is a question of what it means to be “free” when children are largely dependent on their parents. Dependency is practical, in terms of shelter, food, payment for educational and healthcare provision, and also emotional, with needs for love and acceptance [59]. The interviews allude to this, where reasons given for following parents’ desires around consent include living in their home. This effect seems to be magnified where authority figures are more assertive or do not explain that they will not be upset by girls’ choices [37, 60].

#### What constitutes being adequately informed for consent?

By the time of the interviews, understanding of the trial was limited for both girls and parents. It is unclear whether this relates to understanding at time of trial consent, or degradation of understanding over the period between the trial and the interviews. Among both girls and parents, knowledge of key features of the trial, such as it being optional and the practical requirements were better than knowledge of the purpose of the trial or what the vaccine was for, as with other studies [61]. This has implications for how we measure children’s ability to comprehend information for consent, an issue often examined in isolation rather than in comparison to adults. In this way, children are often found not to have capacity to adequately understand information to make consenting decisions [12, 27, 28], although there are exceptions [26, 62]. We assume adults have capacity until proven otherwise, a status not granted to children, but in these interviews, adults and children shared similar gaps in knowledge. Further, adults have been shown to play an essential role in enabling informed adolescents [37], so the implications of relatively “uninformed” adults needs unpacking. Importantly, most interviewees felt sufficiently informed to make the decision and remained comfortable with their decision. Other papers have described varying perspectives on whether participants feel they have enough information to make decisions [15]. One study found that those declining participation were more likely to feel they had insufficient or biased information [53], and it may be that desired information levels and trust are linked.

Strategies suggested for improving understanding of consent in young people include technology usage [53, 61], reminder-recall notices [53] and comprehension checks as used in the DoRIS study [1, 48]. The importance of information sheets has been reinforced [15]. However, these tend to be focused largely on high-income countries, so further work is needed to explore how to best promote understanding in other settings.

### Balancing trust and distrust to make decisions

One of the key themes that emerged from discussions of decision-making was trust and trustworthiness. As with other studies, trust was a required entity for research participation [15, 38, 63]. Trust may be built through active nurturing by trial teams and delegation of power to fieldworkers from research headquarters [63]. However, it is also influenced by factors that are more difficult to alter, such as experiences with other trial teams [43, 63]. Geissler et al. found that the ethics of a malaria vaccine trial in The Gambia were deeply socially ingrained, with an enmeshment of researcher and participant that did not match the divisional nature of international ethical guidelines [43]. He termed this ‘relational’ ethics to account for the importance of “knowing each other” and the dynamism this entailed. This conceptualisation brings interesting insights to these interviews, as it demonstrates how interactions between researchers and families can feed into trust-building and a relational ethics.

The data presented here also showed the importance of, not only trust in the researchers, but sufficient distrust in others to be able to overcome the barrier that rumours formed. This establishes a balance in which trustworthiness of researchers, government and healthcare providers, is set against that of fellow community members and unfavourable opinions they share. Family members often sit in between - both trusted and with acknowledgement that they are not immune to believing false rumours. Interestingly, Fairhead et al. found that narratives of trust seemed to be emphasised by the research staff, whereas parents seemed to see decisions about involvement in terms of dangers and benefits [64]. These interviews suggest that parents and children do balance the dangers, including short-term such as pain, and long-term including rumours about fertility, and benefits, such as protection against cervical cancer [49]. However, rather than this being an alternative to trust, trust forms the foundations of how parents and children weigh these up and which to give credence to.

### Implications for adolescent consent for trials

Both parents and children were clear that they did not advocate for self-consent models. Whilst this may be partly due to familiarity with parental consent, this is also supported by practice and cognitive developmental literature [12]. Engaging a best interests perspective allows us to see benefits of other models, despite a de-prioritisation of autonomy. For example, consent without adequate capacity may be harmful to an adolescent who chooses to participate in something they otherwise would not have [65]. Assent models could be used to protect young people from too much or too little involvement [66], considering the child’s preferred degree of involvement [12] and focusing on the input a child can have to decision-making, irrespective of capacity.

Processes that view either parents or adolescents highly individually are unlikely to provide the space needed for co-creating decisions in the way that both parents and adolescents desired to in the interviews. Whilst assent offers an opportunity for a child to share their opinion at a developmentally appropriate level, it has been criticised for the lack of clear and binding standards and the fact parents continue to make the final decision [1, 35]. Similarly, dual consent models would allow for greater emphasis on the child’s emerging autonomy whilst adding protection in the form of parental consent but challenges arise when there are disagreements [35].

### Strengths and limitations

This project considers the perspectives of both girls and their parents, and paired interviews gave an opportunity for unique discussions, providing rich data on how girls and parents approached and viewed decision-making. It ties together research on the ethics of adolescent vaccine trial involvement with the practicalities of how decisions are made, allowing insight into how to undertake adolescent trials most ethically. Many of the learning points may apply more generally to other adolescent research studies.

There are several limitations to this study. Firstly, the primary aim of this qualitative study was regarding dose reduction acceptability [49]. Exploration of the mechanisms by which decisions are made about vaccination - and the extent to which daughters are involved in this process - was a secondary aim of the study. This meant that the data leant itself to informing some aspects of the research question more than others, limiting the conclusions regarding these elements. For example, further exploration of how disagreements were managed or exactly what “involvement” looked like were not undertaken.

Secondly, interviews were only undertaken with participants that consented to the DoRIS trial, and conclusions must be interpreted in this light. Further research exploring the perspectives of non-consenters would provide valuable insight into how decision-making processes vary across groups and would widen transferability of the findings. This group may be difficult to access, given their previous lack of research participation, so strategies to maximise comfort for participation are key. Similarly, the DoRIS trial only vaccinated girls, and it is unclear how these perspectives may have varied if vaccination of boys was considered.

Finally, interviews are an invaluable tool to explore people’s ideas and experiences but may not reflect behaviours. Actual decision-making practices may vary from what is described in the interviews, particularly surrounding challenging topics like conflict. However, interviews are pragmatic: much of the decision-making surrounding trial consent would be made in private residences, and therefore not amenable to participant observation or other methodologies that explore behaviours in practice. Additionally, interviews provide insight into how individuals think about and interpret the decision-making behind consent and how they feel it should be.

Further work on establishing where experiences of adolescent decision-making and consent for research and clinical practice overlap and diverge will facilitator translation of research knowledge.

## Conclusion

A wide variety of decision-making processes were represented across the interviews, demonstrating the nuance that decision-making models need to be viewed through. Girls take opportunities to exercise their autonomy. Family dynamics, including those between children and adults and between adults, are likely to greatly influence the outcomes. Consent should be reconceptualised away from solely the signing of a form to a relational, negotiated process over time. Relational autonomy provides one tool for this, helping to explain how adolescents enact their autonomy in the context of family and with respect for the emotional experience as well as rational thought.

These insights are of vital importance to public health. Research trials are integral to developing public health interventions, including expanding vaccine programmes. Understanding how decisions are made and who is involved provides opportunities to support these processes in those considering trial participation. This can enable better ethical practice and increased trial recruitment. Exploring these issues for adolescents is essential to ensuring they can benefit from research and be protected from risks. Promoting their involvement through supported means, such as assent or dual-consent, could be an important step to ensuring their opinions are recognised, providing an opportunity for important discussions that may deepen whole-family understanding, and develop future decision-making competence.

## Electronic supplementary material

Below is the link to the electronic supplementary material.

Supplementary Material 1

Supplementary Material 2

Supplementary Material 3

## Data Availability

The datasets used and analysed during the current study may be made available from the corresponding author on reasonable written request. Requests must be accompanied by a defined analysis plan, which will be reviewed by the MITU Data Sharing Committee and senior investigators at London School of Hygiene and Tropical Medicine. Requesting researchers will be required to sign a Data Access Agreement if approval is given.

## Declarations

### Authors’ information

This project was completed as part of a dissertation in partial fulfilment of LF’s MSc Public Health from London School of Hygiene and Tropical Medicine, for which KM was supervising.

## Abbreviations

DoRIS: Dose Reduction Immunobridging and Safety Study of Two HPV Vaccines in Tanzanian Girls
HPV: Human Papillomavirus
WHO: World Health Organization
