# Supplementary material for: Ethical issues in vaccine trial participation by adolescents: qualitative insights on family decision making from a human papillomavirus vaccine trial in Tanzania
Source: BMC Med Ethics. 2024 Nov 20;25:134. doi: 10.1186/s12910-024-01122-z (PMC11577580; doi:10.1186/s12910-024-01122-z)
Supplement: Supplementary file 2 — Supplementary Material 2 [file 12910_2024_1122_MOESM2_ESM.docx]

# DoRIS study Topic Guide for Acceptability study

# Semi-structured interviews with parents of girls who complete the course

**Checklist for Interviewer prior to interview:**

- Ensure there are no other people present (e.g. parents; other adults)
- Introduce yourself
- Remind participant why she has been chosen
- Remind participant about the purpose of the discussion and broadly what you will be discussing
- Ask permission to record (if participant uncomfortable you will need to take notes)
- Ensure informed assent and consent from parents
- Do they have questions?

## Vaccine Course Completer Interview

**Lets start by talking a little bit about yourself** (this is a warm-up question and to set context; should be brief)

**PROBE:**

- Occupation/how you spend your day
- Number of children in household
- Level of education

**We are talking today because your daughter recently completed a course HPV vaccine. Can you tell me what this vaccine is for?**

**PROBE for knowledge of cervical cancer and HPV:**

- Have you heard of cervical cancer? What is it? Who gets it?
- What is HPV? How common? How do you get it? Who is most at risk? Symptoms?
- Is there a link between HPV and cervical cancer?
- What does the vaccine do? Do you know why YOU received the vaccine?

**We are now going to talk a bit about things you/others considered in deciding whether to get the HPV Vaccine**

Can you tell me about the main reasons you accepted to have your daughter vaccinated? Probe ‘what else?’ but don’t offer reasons)

I’m now going to show you a set of cards. Each card shows a different thing that people use to help them decide whether or not to get vaccinated. I’d like you to think about whether you considered these things when deciding whether or not to let your daughter take part in the trial.

Please put the cards into three piles: “ I/we thought about this a lot”, “I/we thought about this a little”, and “I/we didn’t consider this at all”

*Your age*

*How well the vaccine protects against disease (how effective it is)*

*Number of doses (1,2 or 3)*

*Concern about side-effects of vaccine such as pain, headache, fever*

*The risk of getting vaccinated (vaccine safety)*

*Your risk/likelihood of getting Cervical cancer if not vaccinated*

*The severity of the disease (cervical cancer)*

*Views of neighbours and friends about the vaccine*

*The fact that the virus protects against a sexually transmitted disease*

*The views of a professional or someone in authority (doctor, nurse, religious leader)*

*The views of a friend or neighbour or family member*

[Interviewer to discuss the piles with participant, for instance asking about the meaning of cards and why they are in certain piles. Ask specifically about number of doses. For the “thought about this a lot” pile, ask participant to put them in order of importance. Interviewer to take digital photo of completed order of cards on phone]

If you had to pay for the vaccine, would that change your decision?

**Can you tell me about your daughter’s experience of getting the HPV vaccine?**

- How do you feel about your daughter taking part in the study? Probe: Why did you decide your daughter to take part?
  - Did you have any worries or concerns about taking part? (please tell me about them)
  - Who, if anyone, did you talk to about these concerns?
    - What was discussed and did the discussion change how you felt about the vaccination in anyway?
  - Did you, or anyone else, have any concerns about which study group your daughter might be put in?
    - If yes, can you tell me more about those concerns? Did any of those concerns relate to the number of injections she would receive?
  - Do you feel you had enough information from the study team about what it would be like for your daughter to take part in the study? (Probe for reasons behind yes/no answers).
  - Are there any things you would have liked to have had explained to you in more detail?
- How did your daughter experience the HPV vaccine?
  - Probe for expectations beforehand (e.g. about site of vaccination, level of pain, other issues), feelings beforehand (e.g. fear or worry)
  - How was her experience of the vaccination? (ask about pain, swelling and other immediate effects)
  - How did she feel straight afterwards? And since/more recently?
  - Did she receive any doses of the vaccine from outside of this study?

**I’d like to talk now about your preferences in terms of how many doses your daughter received. A dose is an amount of the vaccine that is given at one time.**

**[confirm number of doses, intended and actual]**

- If you were given a choice, how many doses of the HPV vaccine would you prefer your daughter to be given [i.e. how many times would you prefer your daughter to receive it]
  - Why would you prefer this number? (probe about effectiveness, safety, convenience)

**[Q for young women receiving one dose only]**

- When you heard that your daughter would receive one dose of the vaccine, how did you feel?
- Your daughter was given one dose of the vaccine. If she was to receive the vaccine in two or three doses, how would that change (if at all) your feelings about:
  - Whether you wanted her to be vaccinated with the HPV vaccine
  - The safety of the vaccine and risks of receiving it
  - The convenience of being vaccinated
  - Your feelings about whether the vaccine will actually protect her from HPV
  - (If concerns raised previously) Any of the concerns that we’ve talked about so far
- What do you see as the benefits, if any, of taking one dose instead of 2/3? What are the disadvantages, if any?

**[Q for young women receiving 2/3 doses – use 2 or 3 as appropriate to interviewee]**

- When you heard that your daughter would receive 2/3 doses of the vaccine, how did you feel?
- Your daughter received[2/3 doses] of the vaccine. If shewere to receive the vaccine in one dose would that change how you feel about:
  - Whether you wanted your daughter to be vaccinated with the HPV vaccine
  - The safety of the vaccine and risks of receiving it
  - The convenience of being vaccinated
  - Your feelings about whether the vaccine works
  - (If concerns raised previously) Any of the concerns that we’ve talked about so far
- **[3 doses only].** Your daughter was given two injections quite close together. How did you feel about that?

**[All young women]**

- What do you see as the benefits of taking2/3 doses instead of one? What are the disadvantages, if any?
- Why do you think the scientists might try to reduce the HPV vaccine dose from three to one?

**I’d like to talk about how the decision was made to take part in the DoRIS study and receive the vaccination**

- Please tell me about how the decision was made for your daughter to get vaccinated and take part in this study:
  - Who was involved and what did they say?
  - Did all those involved in making the decision agree or were there some differences in opinion? If yes, can you tell me about those?
  - What were your daughter’s views? Did they form part of the discussion in any way?
  - If the decision had been up to your daughter, might she have made a different choice?
  - Did you feel you had enough information to make your decision? If no, what else would you like to have known more about?
  - What do you think is the best way for families to make decisions about adolescent vaccination? (probe: parents just decide, parents consult then decide, daughter should get to decide)
  - Thinking about other decisions that have been made about your daughter’s wellbeing or future (e.g. range from how she spends hertime, subject choices at school, future plans) – how are these decisions made in your home? (probe for level of consultation and involvement, level of autonomy and control, expectations of parent and daughter)

**Can you tell me what you think of the HPV vaccine?**

PROBES

- Do you think it matters whether girls of your daughter’s age get this HPV vaccine?
  - Why/why not?
  - What do you see as the advantages/disadvantages?
  - What do you think is the ideal age for girls to be given the vaccine?
  - Do you think boys should receive the vaccine?
  - Do you think the vaccine should be given to all girls or just those who are sexually active?
  - Any other thoughts, beliefs or opinions about the HPV vaccine that you are happy to share?
- To what extent do you think that HPV and cervical cancer are risks to your daughter? (ask about risk of HPV and risk of cancer separately; after discussing it, ask participant to give numerical score with 1 being ‘no risk at all’ and 10 being ‘almost certain to happen’)
  - Can you tell me why you think she is/is not at risk?
  - Do you think her level of risk might change in future? How and why?
  - To what extent do you think that getting this vaccine will protect her from the risks you’ve described?
- We’ve talked about what you know about the HPV Vaccine. How did you learn these things?
  - Ask about sources and what was discussed.
  - Do you feel you know enough or are there more things you’d like to know? (if yes, what)

**[section below to be covered if time allows]**

**Can you tell me what others think of the HPV vaccine?**

- Did you hear any stories or concerns about the HPV vaccine among people that you know such as health workers, neighbours and friends, community leaders?
  - If yes, what did you hear?
  - And what do you think about those views?
  - How, if at all, do these views affect your own views on the HPV vaccine?
- And did you hear any views about the HPV vaccine expressed on television and radio?
  - If yes, what did you hear?
  - And what do you think about those views?
  - Did they affect how you felt about getting vaccinated in any way?
- Have you heard any other stories or concerns (or rumours) about the HPV vaccine in your community, that we’ve not discussed above?
  - If yes, please tell me about these concerns and who has them?
  - To what extent do you agree with these concerns and why?
- Whose opinions on vaccination do you value and trust most and why?
  - What about others, who is seen as most influential in your community when it comes to beliefs about vaccines?
  - Did any of the concerns we’ve talked about have an influence on how you felt about your daughter taking part in this study?
- People have very different opinions of vaccinations; I’d like to know what you think of these opinions.
  - Some people say that vaccines are dangerous and that they make people ill. What is your view of that?
  - Some people say that vaccines often don’t work. What is your view of that?
  - Some people say that giving the HPV vaccine to young girls will encourage them to be sexually active. What is your view of that?
  - Some parents say that there is no need for their daughter to be vaccinated as they will not be at risk of HPV. What is your opinion of that?

**Do you have any other thoughts or concerns about the vaccine, or about your experience of taking part that you’d like to discuss with me?**

**Thank You very much for your time and for sharing your opinions with us.**

[END]
